# Peer Review: Publication’s Gold Standard

**Published:** 2012-03-01

**Authors:** Kelley D. Mayden

**Affiliations:** Ms. Mayden is an oncology nurse practitioner at Southwest Virginia Cancer Center in Norton, Virginia.

The dissemination of valuable and novel scientific information provides the pulse for biomedical publishing. Scientific journals catalog the contributions, thoughts, and opinions of researchers, investigators, and experts in the field. Authors consider the reputation and quality of a journal prior to submitting a manuscript for consideration. It is reasonable to think that readers also consider journal prestige as a factor in journal selection. The prestige of a journal depends on the validity, usefulness, and quality of the articles published. This article will define and examine the peer-review process as well as explore the roles and responsibilities of the peer reviewer.

## The Peer-Review Process

Aside from its use in scientific journals, peer review is the process by which grants are allocated, academics are promoted, textbooks are written, and Nobel prizes are won (Smith, 2006). A publication that has been peer reviewed gains respectability and acceptance and is considered a relevant contribution to the field. Publication in a peer-reviewed journal is an important criterion for admissibility of scientific evidence in courts of law (Kumar, 2009). The basis of the peer-review process is the acceptance of written investigational findings from an author or group of authors that are then forwarded to a group of experts (referees) in the field for assessment of their quality, accuracy, relevance, and novelty (Shuttleworth, 2009). Traditionally, these experts are not paid for their opinions and are not part of an editorial staff.

**Figure 1:**
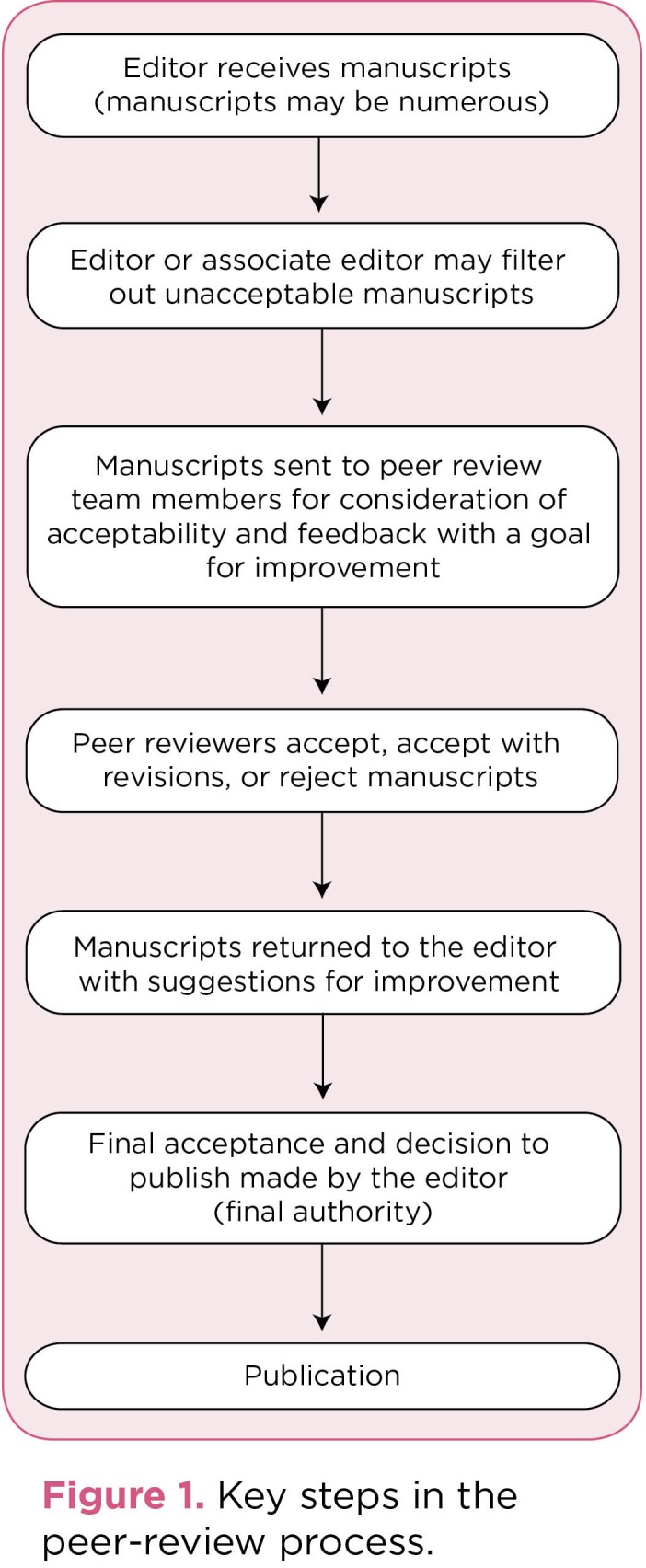
Figure 1. Key steps in the peer-review process.

The goal of peer review is to determine if an article should or should not be published and to improve the article before publication (Neale & Bowman, 2006). It is a process that entails filtering out manuscripts that are misleading, irrelevant, inaccurate, or that contain potentially harmful content (Kumar, 2009). Once the peer-review process is complete (see Figure 1), the editor of a journal bears responsibility for its content and may choose to agree or disagree with the opinions of the reviewers (Garmel, 2010).

## Limitations

Despite its acceptance as a critical part of quality control, peer review is not a perfect process. In 2003, *The Cochrane Collaboration* published a review concluding that there is little evidence to support the use of editorial peer review as a mechanism to ensure quality of biomedical research, despite its widespread use and costs (Jefferson, Rudin, Brodney Folse, & Davidoff, 2007). There are few published, randomized controlled studies relating to peer review; therefore it remains ill-defined.

The peer-review process can be time consuming, costly, subject to reviewer bias, and inept at identifying fraudulent manuscripts. A well-known example of the failure of peer review is the publication of two fraudulent papers by Hwang Woo-Suk concerning stem cell research in the journal *Science* (Kumar, 2009).

In addition, there are no agreed-upon evidence-based guidelines as to what constitutes a qualified reviewer. A study examining the relationship of previous training and experience of journal peer reviewers to subsequent review quality determined that no identifiable types of formal training or experience predicted reviewer performance. The authors suggest that journals implement routine review rating systems to periodically monitor the quality of their reviews (Callaham & Tercier, 2007).

Traditionally, the peer-review process has been conducted anonymously, with author and reviewer identities masked during the review process. Although this may protect reviewers from author demands and retaliation, reviewer anonymity is being debated and is under increasing scrutiny (Garmel, 2010; Leek, Taub, & Pineda, 2011). Early evidence supporting blind peer review (McNutt, Evans, Fletcher, & Fletcher, 1990) was later challenged by studies suggesting that such a practice made no editorially significant difference to review quality, publication recommendation, or time taken to review, but did increase the probability of reviewers declining to review (van Rooyen, Godlee, Evans, Smith, & Black, 1998; Justice, Cho, Winker, Berlin, & Rennie, 1998; van Rooyen, Godlee, Evans, Black & Smith, 1999). It is possible that an open process may increase cooperation between reviewers and authors and lead to a decreased risk of reviewing errors (Leek, Taub, & Pineda, 2011).

Some journals have already considered transition to open peer review. In 1999, the *British Medical Journal* adopted an open (signed) review system that remains in place today. Most recently, the journal has examined the effect of notifying reviewers that their signed reviews might be posted on the web. Their conclusion was that alerting peer reviewers that their signed reviews might be available in the public domain on the journal’s website had no important effect on review quality but was associated with a high refusal rate (van Rooyen, Delamothe, & Evans, 2010). Other journals such as *Nature* and *The Public Library of Science* are revising old review criteria, creating open access, and examining public review (Editors of *The New Atlantis*, 2011).

One study examined the effects of adding a statistical peer reviewer and using a checklist of manuscript quality. The study showed a positive effect when a statistical reviewer was added to the field-expert peers, but no statistically significant positive effect was suggested by the use of reporting guidelines (Cobo et al., 2007). Additional alternative methods of peer review such as open peer review without suppression of publication, postpublication review, a hybrid system (traditional with postpublication review), author-suggested peer review, author model of peer review, and peer review consortia have been discussed and explored in the literature (Kumar, 2009).

## Reviewer Responsibilities

However ill-defined it may be, the peer-review process is still the gold standard that will continue to drive scholarly publication. Understandably, a large part of the responsibility for the success or failure of the peer-review process depends upon peer reviewers. A peer reviewer should be both a scholar and a scientist with complex analytical skills, which allows for the critical analysis of data in the interest of improved outcomes (Bearinger, 2006).

Peer review can be time consuming and laborious; therefore, accepting the responsibility of peer review requires commitment on the part of the reviewer. It should be viewed as a professional responsibility, not to be taken lightly, given that the end result determines what is relevant, in print, to a specific body of knowledge. Just as editors and journals respect their reviewers, often acknowledging their contributions publically, reviewers should respect the editor and the journal by producing a quality of work that is consistent with the journal’s reputation and integrity.

Just as a surgeon would prepare for surgery, a reviewer must prepare for a review. First, it is important to understand a selected journal’s mission and review criteria as they will be incorporated into manuscript review. Once an invitation to review is accepted, reviewers normally agree to complete the assigned manuscript review within a specified time frame. This is not only important to journals and editors who have publication deadlines, but to authors who eagerly await news of acceptance or rejection. Time is especially important in cases where the author is asked to consider recommended revisions prior to a final decision of acceptance or rejection. Second, reviewers must maintain confidentiality; using any information gained for self-interest or extracurricular professional discussion is unethical.

Given that a reviewer’s authority to recommend a manuscript’s acceptance or rejection carries weight with an editor’s final publication decision, careful consideration of the manuscript and each individual section is required prior to any such recommendation. A fair analysis requires a reviewer to have undisturbed focus, a discerning eye for detail, and knowledge of appropriate sectional content (see Table 1). It is important to consider if the information is accurate, understandable, valid, useful, and transparent. Grammar is important, and errors can be pointed out; however, the main concern for the reviewer is relevancy of manuscript content.

**Table 1:**
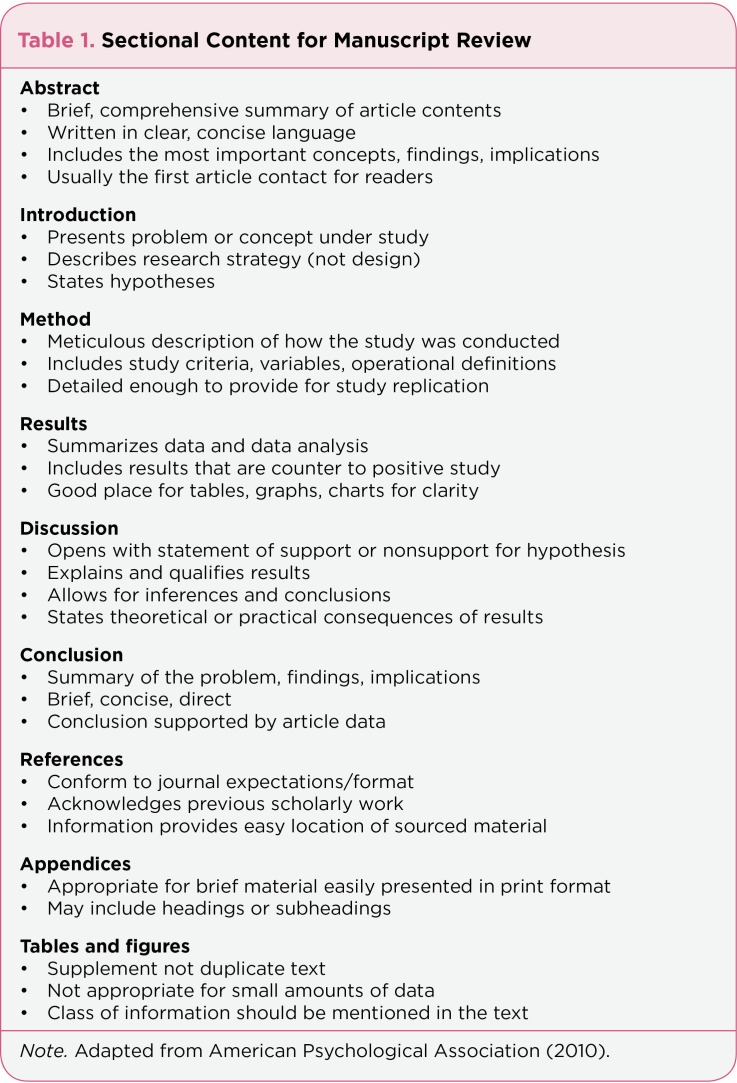
Table 1. Sectional Content for Manuscript Review

Table 2 provides a list of important questions to consider when reviewing a manuscript. A helpful resource to guide review is the CONSORT Statement. Updated in 2010, it provides guidance for reporting all randomized controlled trials (CONSORT, 2010). An additional resource is the EQUATOR Network (2012), an international initiative that seeks to improve the reliability and value of medical research literature by promoting transparent and accurate reporting of research studies.

**Table 2:**
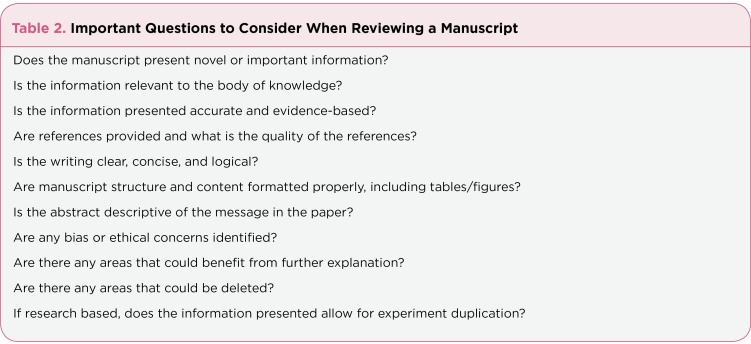
Table 2. Important Questions to Consider When Reviewing a Manuscript

All reviewers are subject to bias. Gender, patriotism, and linguistic preference have been shown to affect peer review (Kumar, 2009). Reviewers are more likely to favor manuscripts that are clearly written, are creative, demonstrate positive results, and have interesting titles, meanwhile rejecting manuscripts with negative results, multiple errors, and seasoned information (Garmel, 2010). It is possible that senior reviewers may reject their juniors; manuscripts from more prestigious institutions may be more readily accepted than those from lesser-known institutions (Kumar, 2009). Reviewers are responsible for disclosing biases that may hinder an impartial and balanced review. Lack of expertise in an area may not hinder review as useful comments may still be collected, but in this circumstance, the editor should be informed that a lack of expertise exists (Garmel, 2010).

Once the review is complete, reviewers offer scholarly input with the intent to improve the manuscript. Feedback should be constructive and the critique professional and positive. When a reviewer provides feedback that enables authors to revise and resubmit a publishable paper, the peer-review process is working as intended (Bearinger, 2006). Length of the review is not as important as detailed suggestions for improvement. The review should begin with a recommendation for rejection, acceptance with minor revisions, or acceptance with major revisions. The reviewer should comment on the manuscript as a whole, then provide input on each individual section. Suggestions should be clear and provide direction. Comments should be detailed enough to assist authors with revisions but not so detailed that the manuscript is rewritten (Garmel, 2010). Reviewers should remember to comment on the appropriateness of the abstract and be certain it mirrors the content of the manuscript.

Reviewing provides an opportunity for learning and gaining exposure to cutting-edge research (Bearinger, 2006). Reviewing is a skill that requires critical thinking; it will improve with time, practice, personal research, and writing. A good reviewer is competent, knowledgeable, unbiased, objective, punctual, consistent, ethically sound, constructive, and maintains confidentiality (Garmel, 2010; Kumar, 2009).

## Feedback

Reviewers, like authors, can benefit from feedback; they should welcome input from editors and experienced colleagues. Feedback is important for both new and seasoned reviewers. Editors at a specialty journal in the top 11% of the Institute of Scientific Information’s bibliographic database (ranked by number of citations) performed a 14-year longitudinal study designed to evaluate change in the review quality of individual peer reviewers. The study found that over time most journal peer reviewers received lower quality scores for article assessment. Proposed reasons were cognitive changes, competing priorities, or escalating expectations (Callaham & McCulloch, 2011). Although it is not common practice, results such as these suggest that ongoing self-evaluation by the reviewer and validated reviewer evaluation on the part of the editor are important factors for ensuring quality peer review.

Reviewing is a professional privilege, and reviewers are advised to remember they are representing a journal and have responsibilities to authors (see Table 3), editors (see Table 4), and readers (see Table 5). Perhaps most importantly, reviewers are accountable to the medical community and the scientific body of knowledge impacted by their reviews.

**Table 3:**
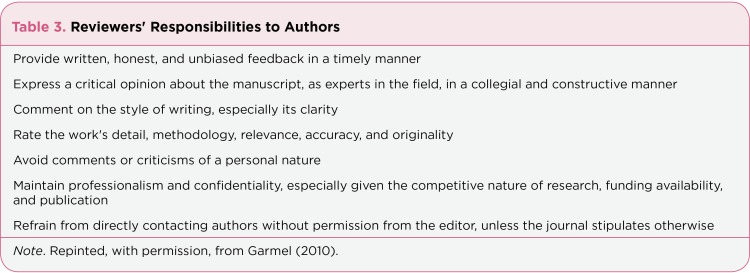
Table 3. Reviewers' Responsibilities to Authors

**Table 4:**
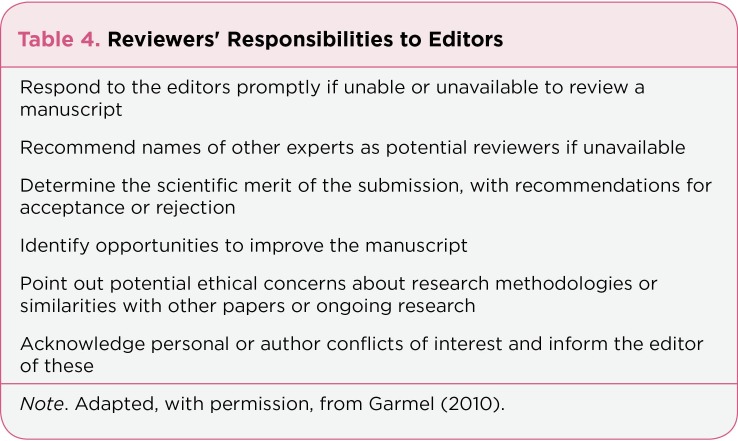
Table 4. Reviewers' Responsibilities to Editors

**Table 5:**
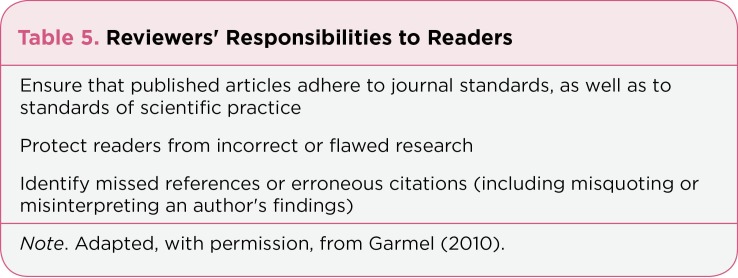
Table 5. Reviewers' Responsibilities to Readers

## Conclusion

While it is not a perfect process, traditional peer review remains the gold standard for evaluating and selecting quality scientific publications. Additional research and the development of evidenced-based guidelines are needed to govern this process, which is expected to evolve in the future. Peer review is both an art and a science largely dependent on the quality of its review body. Competent peer reviewers are experts in their field accountable to authors, editors, readers, and the medical community. Peer reviewers act as advocates, or referees, for authors and enable editors to make quality publication decisions. Peer review is a professional privilege and responsibility that directly impacts what is accepted as important to a body of knowledge. Although the peer-review process can be time consuming and underappreciated, rewards such as mentorship, learning, exposure to cutting-edge research, and personal development make it a worthwhile investment.
